# What is the incidence and clinical significance of dry eye disease in patients treated with immune checkpoint inhibitors? A systematic review and meta‐analysis of ocular immune‐related adverse events

**DOI:** 10.1111/aos.70058

**Published:** 2026-03-09

**Authors:** Kai‐Yang Chen, Hoi‐Chun Chan, Chi‐Ming Chan

**Affiliations:** ^1^ Department of General Medicine Chang Gung Memorial Hospital (Linkou Branch) Taoyuan Taiwan; ^2^ School of Pharmacy China Medical University Taichung Taiwan; ^3^ Department of Ophthalmology Cardinal Tien Hospital New Taipei City Taiwan; ^4^ School of Medicine Fu Jen Catholic University New Taipei City Taiwan

**Keywords:** cancer immunotherapy, dry eye disease, immune checkpoint inhibitors, incidence, lacrimal dysfunction, ocular adverse events, risk factors

## Abstract

**Purpose:**

Immune checkpoint inhibitors (ICIs) have transformed cancer therapy but may cause immune‐related adverse events (irAEs), including dry eye disease (DED). This study aimed to quantify the incidence of ICI‐associated DED and to evaluate factors contributing to variability across studies.

**Methods:**

A systematic review and meta‐analysis was conducted following the Preferred Reporting Items for Systematic Reviews and Meta‐Analyses (PRISMA) guidelines and was registered in the International Prospective Register of Systematic Reviews (PROSPERO; CRD420251091266). PubMed, the Cochrane Library, and Web of Science were searched from inception through September 13, 2025. Eligible studies included adults (≥18 years) treated with ICIs (anti‐programmed cell death protein 1 (PD‐1), anti‐programmed death‐ligand 1 (PD‐L1), anti‐cytotoxic T‐lymphocyte–associated protein 4 (CTLA‐4)) reporting DED outcomes with extractable ICI‐exposed denominators. Two independent reviewers screened studies, extracted data, and assessed risk of bias using the Newcastle–Ottawa Scale (observational studies), ROBINS‐I (non‐randomized interventional studies), and Cochrane RoB 2 (randomized controlled trials). Pooled incidence and 95% confidence interval (CI) estimates were calculated using random‐effects models on the logit‐transformed scale and back‐transformed to proportions. Subgroup analyses evaluated ICI class, combination therapy, and tumour type.

**Results:**

Thirteen studies were included. The pooled incidence of DED among ICI‐treated patients was 2% (95% CI: 1%–3%), with marked heterogeneity across studies (0.2%–65%), likely reflecting variation in surveillance intensity, diagnostic criteria, and ascertainment approaches. Combination ICI therapy (CTLA‐4 plus PD‐1) demonstrated a numerically higher pooled incidence (25%) than monotherapies (CTLA‐4: 5%; PD‐1: 13%; PD‐L1: 15%), although subgroup differences were not statistically significant (*p* = 0.18); these regimen‐stratified estimates were derived from a limited subset of studies with extractable regimen‐specific denominators and heterogeneous surveillance intensity and therefore are not directly comparable to the overall pooled incidence. Lung cancer cohorts showed higher observed rates (41%) compared with melanoma (4%) and renal cancer cohorts (32%) (*p* = 0.05); however, these apparent subgroup differences may reflect not only tumour‐ or regimen‐specific biology, but also systematic differences in surveillance intensity, access to ophthalmic assessment, and referral enrichment across cohorts, which may materially influence observed event rates. Mechanistic findings were consistent with immune‐mediated lacrimal dysfunction, with abnormal Schirmer’s test results reported in 62% of symptomatic cases. Most DED events were mild and managed with topical therapy, while a minority required escalation to systemic immunomodulation.

**Conclusion:**

DED occurs in approximately 1 in 50 ICI‐treated patients in pooled estimates from heterogeneous study designs, although reported incidence varies substantially across study designs and ascertainment approaches. Combination therapy and ICI‐treated lung cancer cohorts demonstrated numerically higher DED rates, but these subgroup estimates should be interpreted cautiously given potential differences in referral patterns and diagnostic intensity. Incidence variability highlights the need for standardized ocular assessment. High‐risk patients should be proactively monitored to maintain quality of life and treatment adherence.

## 1 INTRODUCTION

Regarded as the cornerstone of modern oncology, immune checkpoint inhibitors (ICIs) constitute a cluster of cancer immunotherapies designed to boost anti‐cancer immune responses through targeted action on immunological receptors on the surface of T‐lymphocytes (Shiravand et al., 2022; Wojtukiewicz et al., 2021). This cluster includes anti‐programmed cell death protein 1 (PD‐1), anti‐programmed death‐ligand 1 (PD‐L1) and anti‐cytotoxic T‐lymphocyte‐associated protein 4 (CTLA‐4) therapies (Meng et al., 2025). By enhancing the immune system's ability to target cancer cells, ICIs have significantly improved patient outcomes (Zamani & Šácha, 2025). Nonetheless, the widespread utilization of ICIs has been associated with a spectrum of immune‐related adverse events (irAEs), which have been shown to affect multiple organ systems, including the ocular surface (Lerch & Ramanathan, 2025; Wu, Yakobi, et al., 2024; Zhou & Wei, 2021).

According to prior reports by Dalvin et al. and Antoun et al., almost 1% of patients treated with ICIs have reported ocular side effects (Antoun et al., 2016; Dalvin et al., 2018). However, the literature investigating this association has highlighted incidence rates of 2.8% and 4.3% (Bomze et al., 2022; Sun et al., 2020). Among these events, dry eye disease (DED), characterized by tear film instability, ocular surface inflammation and visual discomfort, has emerged as a notable but understudied complication of ICI therapy (Messmer, 2015). DED can significantly impair quality of life, leading to ocular discomfort, foreign body sensation and visual disturbances. While typically managed with topical therapies without requiring ICI interruption, its symptomatic burden can affect patient well‐being and, in rare severe cases, may complicate treatment adherence. Discontinuation of ICIs is generally reserved for severe, sight‐threatening ocular irAEs (e.g. uveitis and retinopathy), rather than for isolated DED (Aragona et al., 2025; Narendra et al., 2025; Uchino & Schaumberg, 2013).

Despite increasing reports of ocular irAEs, the incidence of DED in patients receiving ICIs remains poorly characterized, with studies reporting variable rates due to differences in study design, patient populations and diagnostic criteria. A comprehensive synthesis of available evidence is needed to quantify the risk of DED associated with ICI therapy and to inform clinical management strategies. To address this gap, we conducted a systematic review and meta‐analysis to assess the incidence of DED in patients treated with ICIs. This study aims to provide a pooled estimate of DED incidence, explore potential sources of heterogeneity (e.g. ICI type, cancer type or treatment duration) and evaluate the quality of evidence. By elucidating the burden of DED in this population, our findings seek to guide clinicians in monitoring and managing ocular complications, ultimately improving patient care and outcomes in the era of immunotherapy.

## 2 MATERIALS AND METHODS

### 2.1 Study design

This systematic review and meta‐analysis was conducted in accordance with the Preferred Reporting Items for Systematic Reviews and Meta‐Analyses (PRISMA) guidelines. The protocol was registered with PROSPERO (International Prospective Register of Systematic Reviews; registration number: CRD420251091266).

### 2.2 Eligibility criteria

#### 2.2.1 Inclusion criteria

Studies were included if they met the following criteria:

*Population:* Adult patients (≥18 years) treated with ICIs (e.g. anti‐PD‐1, anti‐PD‐L1 or anti‐CTLA‐4 agents) for any malignancy.

*Intervention/Exposure:* anti‐PD‐1, anti‐PD‐L1 or anti‐CTLA‐4 agents, including nivolumab, pembrolizumab, ipilimumab, atezolizumab, durvalumab and avelumab for any malignancy.

Outcomes:

- *Primary outcomes:* Studies reporting incidence of DED, defined as the number of unique affected patients divided by the total number of unique patients receiving ICIs. Pharmacovigilance disproportionality analyses were summarized descriptively and were not pooled for incidence estimation because they do not provide a valid denominator of ICI exposed patients.
- *Secondary outcomes*: Studies reporting severity (e.g. Common Terminology Criteria for Adverse Events, CTCAE), clinical features (e.g. Schirmer’s test and Ocular Surface Disease Index, OSDI) and management strategies.

Study types:

- Randomized controlled trials (RCTs), cohort studies, case–control studies, case series (≥10 patients) and pharmacovigilance analyses (e.g. the US Food and Drug Administration Adverse Event Reporting System (FAERS) and the World Health Organization (WHO) global database of individual case safety reports (VigiBase)).
- Notably, only studies with explicit reporting of DED (not just ‘ocular AEs’) were included.
- Studies published in English that provide sufficient data to calculate the incidence of DED were included.
- Case series, particularly those derived from tertiary referral settings, were included primarily to characterize the clinical phenotype, diagnostic evaluation, and management of ICI‐associated DED; however, such studies were not assumed to be population‐representative for estimating incidence in unselected ICI‐exposed populations due to potential referral bias.

#### 2.2.2 Exclusion criteria

- Non‐human studies, reviews, editorials and conference abstracts without complete data.
- Case reports (<10 patients) unless pooled in a larger analysis.
- Studies without a clear denominator (e.g. missing sample size).

### 2.3 Search strategy

A comprehensive literature search was conducted from database inception to 13 September 2025, across PubMed, Web of Science and the Cochrane Library, with no restrictions on the lower date limit. The search strategy combined Medical Subject Headings (MeSH) and free‐text terms, including ‘immune checkpoint inhibitors’, ‘programmed cell death protein 1’, ‘PD‐L1’, ‘CTLA‐4’, ‘dry eye disease’, ‘ocular adverse events’ and ‘immune‐related adverse events’. The full search strategy is provided in Appendix A. Additional studies were identified by manually screening reference lists of relevant articles and conference abstracts from major oncology meetings (e.g. ASCO and ESMO) published within the last 15 years.

### 2.4 Study selection and data extraction

Two independent reviewers (K. Y. C. and H. C. C.) screened titles and abstracts for eligibility, followed by a full‐text review of potentially relevant studies. Discrepancies were resolved through discussion or consultation with a third reviewer (C. M. C.). Data were extracted using a standardized form, including study characteristics (e.g. design, sample size, publication year), patient demographics (e.g. age, sex, cancer type), ICI type and regimen, DED diagnostic criteria, incidence of DED (number of events and total patients) and follow‐up duration. When necessary, authors of included studies were contacted for additional data.

### 2.5 Risk of bias assessment

The methodological quality of included studies was assessed using the Newcastle–Ottawa Scale (NOS) for observational studies and the Cochrane Risk of Bias 2 (RoB 2) tool for randomized controlled trials. Moreover, the Risk Of Bias In Non‐randomized Studies of Interventions (ROBINS‐I) tool was used to assess non‐randomized studies of interventions included in the review. Two reviewers (K. Y. C. and H. C. C.) independently evaluated each study, with discrepancies resolved by consensus. Studies were classified as having low, moderate or high risk of bias based on predefined criteria.

### 2.6 Statistical analysis

The primary outcome was the pooled incidence of DED in patients treated with ICIs, calculated as the proportion of patients with DED among the total treated population. Incidence rates were pooled with 95% confidence intervals (CIs). To avoid impossible confidence interval bounds for proportions, pooled estimates were computed on the logit‐transformed scale and back‐transformed to the proportion scale. Heterogeneity was assessed using the *I* ^2^ (*I*‐squared statistic). An *I* ^2^ value >50% indicated substantial heterogeneity, which is consistent with the commonly cited thresholds in the Cochrane Handbook. A random‐effects model (DerSimonian‐Laird method) was selected a priori to account for anticipated between‐study variability and to provide a more conservative pooled estimate. Subgroup analyses were planned to explore sources of heterogeneity, including ICI type (e.g. anti‐PD‐1 vs anti‐PD‐L1 vs anti‐CTLA‐4), cancer type and DED diagnostic method. Publication bias was evaluated using funnel plots if at least 10 studies were included. Sensitivity analysis, specifically the leave‐one‐out analysis, was conducted to examine the robustness of the pooled estimates. All statistical analyses were performed using STATA version 18.0 (StataCorp, College Station, TX). A two‐sided *p* < 0.05 was considered statistically significant.

## 3 RESULTS

### 3.1 Literature search

**FIGURE 1:**
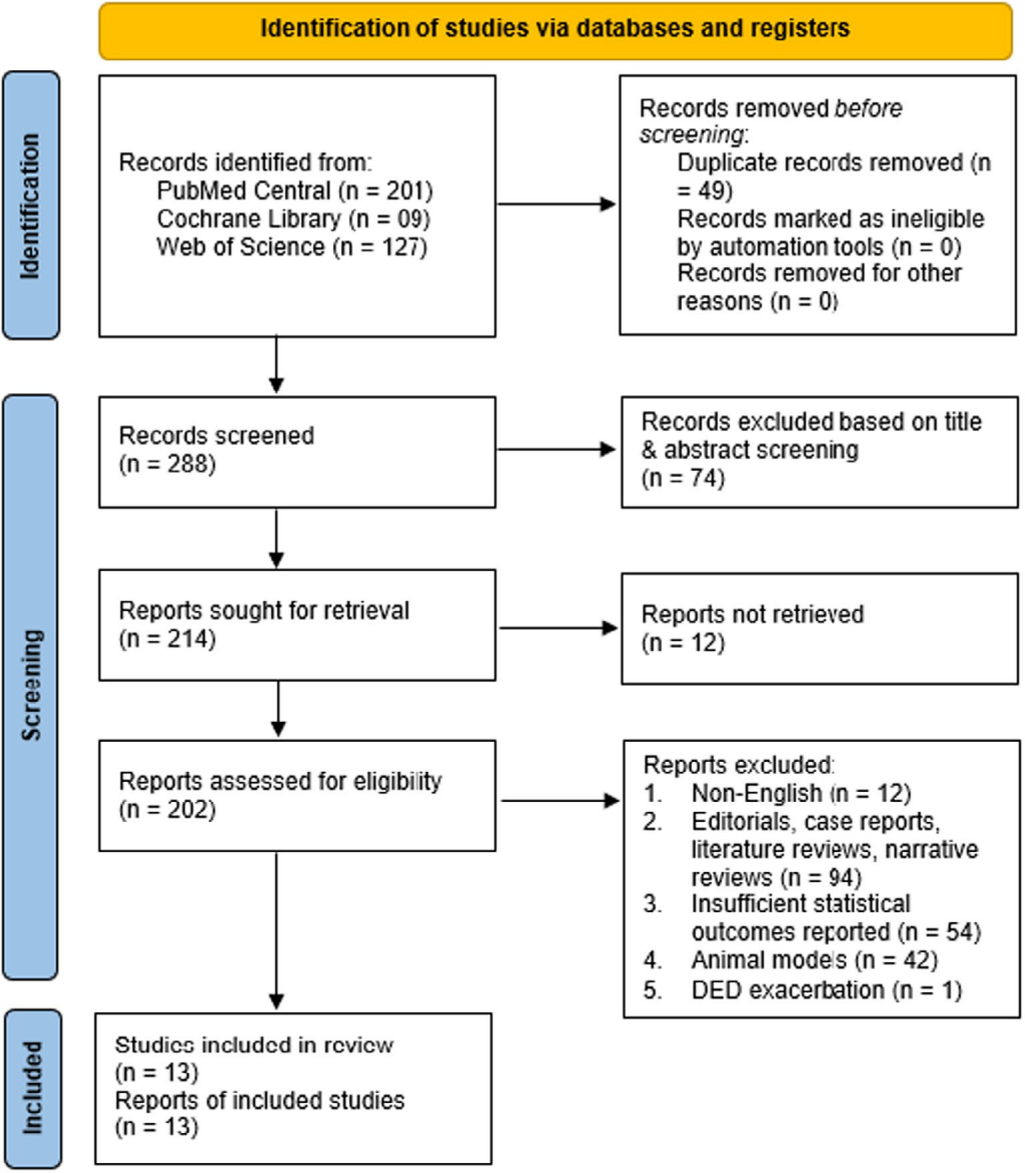
A Preferred Reporting Items for Systematic Reviews and Meta‐Analyses (PRISMA) flow chart summarizing the search process in identifying studies from databases and registers.

This PRISMA flow diagram summarizes the identification, screening, and selection process for the included studies. In the identification phase, a total of 337 records were retrieved from database searches, including 201 records from PubMed, 9 records from the Cochrane Library, and 127 records from Web of Science. Prior to screening, 49 duplicate records were removed, while 0 records were marked as ineligible by automation tools and 0 records were removed for other reasons, resulting in 288 unique records that proceeded to the screening stage. During title and abstract screening, 74 records were excluded based on insufficient relevance to the research question, leaving 214 reports sought for retrieval. Of these, 12 reports could not be retrieved, yielding 202 reports that were successfully assessed for full‐text eligibility. Following full‐text evaluation, 189 reports were excluded for predefined reasons (Figure 1). Ultimately, 13 studies met all eligibility criteria and were included in the systematic review and meta‐analysis (Figure 1).

### 3.2 Characteristics of included studies

Thirteen high‐ to moderate‐quality studies were included in the systematic review and meta‐analysis following the predefined screening and study identification process. All studies were published between 2012 and 2022, investigating DED incidence in adult patients treated with ICIs for various malignancies. Notably, the incidence of DED varied widely across the included studies. This variability was attributed to the heterogeneity in sample sizes, ranging from 11 to 7727 participants, and study designs.

Overall, the included studies displayed heterogeneous study designs, with retrospective study designs predominant (8 out of 13). These retrospective studies included retrospective database analysis of the FAERS database (Fang et al., 2019), the Cancer Therapy Evaluation Program (CTEP) database (Young et al., 2021), and the International ImmunoCancer Registry (ICIR) database (Ramos‐Casals et al., 2019); retrospective cohort studies (Fortes et al., 2021; Mazharuddin et al., 2022; Zimmer et al., 2016), and retrospective case series (Cappelli et al., 2017; Noble et al., 2020). The review included one prospective cohort study (Warner et al., 2019). Non‐randomized clinical trials, including a phase 1 non‐randomized dose‐escalation trial (Hodi et al., 2014), a phase 1 expansion cohort trial (Hamid et al., 2013) and an open‐label multicentre, dose‐escalation trial, were included in the review. Only a single RCT (Weber et al., 2016) assessed the incidence of DED in patients treated with ICIs. Only studies with an extractable denominator of total ICI‐treated patients and patient‐level numerators for DED were included in incidence pooling, whereas pharmacovigilance signal detection studies were summarized descriptively.

The included studies assessed a wide range of ICIs, including PD‐1 inhibitors such as Nivolumab and Pembrolizumab/lambrolizumab; PD‐L1 inhibitors, including Durvalumab, Atezolizumab and Avelumab. Moreover, cytotoxic T‐lymphocyte‐associated protein‐4 inhibitors (CTLA‐4), specifically ipilimumab, were assessed for the incidence of DED. Combination therapies involving ipilimumab and bevacizumab (Hodi et al., 2014) and several non‐specified combinations were also noted throughout the included studies. Key findings highlighted the variability in DED incidence across different ICIs. Nivolumab was most frequently associated with DED, with a high frequency of reports in pharmacovigilance data and higher rates in some clinical cohorts, and it reported the highest incidence in multiple studies (Fang et al., 2019; Ramos‐Casals et al., 2019), while atezolizumab showed no DED cases in one study (Fang et al., 2019). PD‐1 and PD‐L1 inhibitors collectively demonstrated a higher propensity for ocular surface adverse events compared to CTLA‐4 inhibitors, though combination therapies (e.g. ipilimumab/nivolumab) also contributed to DED onset. Severe symptoms, such as photophobia and abnormal Schirmer test results (≤5 mm), were reported in some cases (Ramos‐Casals et al., 2019; Warner et al., 2019), underscoring the clinical significance of DED in ICI‐treated patients.

Other notable ocular adverse events included uveitis, scleritis and keratitis, often co‐occurring with DED. Most studies emphasized that DED could be managed without discontinuing ICI therapy, though severe cases required multidisciplinary intervention. The wide range in reported incidence (0.2%–65%) likely reflects differences in diagnostic criteria, reporting methods and population characteristics, suggesting the need for standardized monitoring protocols in future research (Table 1).

**TABLE 1:** Characteristics of selected studies that met the inclusion criteria.

Study ID	Study design	Sample size (*N*)	ICIs evaluated	Key findings on dry eye disease (DED)	DED ascertainment	DED diagnosis	DED assessment	Other ocular adverse events
Brahmer et al. (2012)	Open‐label multicentre, dose‐escalation clinical trial. NCT00729664	207	Anti‐PD‐L1 (BMS‐936559)	Dry eye was reported in 3% of patients (attributed to treatment)	Prospective safety monitoring in a multicentre Phase 1 dose‐escalation/expansion trial of the anti‐PD‐L1 antibody (BMS‐936559)	No protocol‐driven ophthalmic‐specific testing (e.g. Schirmer's test, OSDI questionnaire, or mandatory ophthalmologist evaluation) was reported. Diagnosis and grading relied on clinical assessment	Adverse events (AEs) were identified through clinical examination and patient reporting, then graded by investigators using the CTCAE v3.0. ‘Dry eye’ was a pre‐specified ‘adverse event of special interest’ due to potential immune‐related causes	Uveitis, endophthalmitis, blurred vision
Cappelli et al. (2017)	Retrospective case series (single centre)	13 patients (83% male)	Nivolumab, ipilimumab, combination therapy	Sicca syndrome (dry mouth) in four patients (31%); dry eye symptoms were less severe	Part of a retrospective clinic‐based case series of rheumatic immune‐related adverse events (iRAEs). Patients were referred by oncologists for new symptoms	‘Severe dry eyes’ as determined by an ophthalmologist's evaluation (specific criteria not detailed)	Salivary gland ultrasound performed in one patient, showing Sjögren's‐like hypoechoic foci. No routine Schirmer's test or other quantified dry eye testing was reported	Inflammatory arthritis, colitis and pneumonitis. No anti‐Ro/La antibodies in sicca patients
Fang et al. (2019)	Retrospective disproportionality analysis (FAERS database)	113 ocular AEs	Atezolizumab, ipilimumab, nivolumab and pembrolizumab	Overall, 13 DED cases were reported. Specific ICI analysis showed Nivolumab had the highest incidence of ICIs (*n* = 11), with Ipilimumab and Pembrolizumab reporting one event each. Atezolizumab did not display any incidence of DED	Disproportionality (pharmacovigilance signal detection) analysis of spontaneous reports in the U.S. FDA Adverse Event Reporting System (FAERS) database (2003–2018)	Unspecified	Cases were identified using the standardized term ‘dry eye’ (or ‘dry eye syndrome’) within the database's reporting structure. No clinical assessment, objective testing	Uveitis, ocular myasthenia, and eye inflammation
Fortes et al. (2021)	Retrospective cohort (single centre)	996 patients (28 with ocular AEs)	Pembrolizumab, nivolumab, atezolizumab, avelumab, durvalumab and ipilimumab/nivolumab	Dry eye is the most common ocular AE (57%, 16/28). PD‐L1 inhibitors had higher ocular surface AEs	Retrospective review of patients seen by an eye care provider (ophthalmologist or optometrist) after ICI initiation, identified via ICD‐10 billing codes and medical record screening	Clinical diagnosis of ‘dry eye syndrome’ made by the evaluating eye care provider	No protocol‐defined objective testing was mandated; diagnosis and management were based on standard clinical practice	Uveitis (14%), episcleritis and serous retinal detachment. Most managed without stopping ICIs. NOTE: The study reported a higher frequency of ocular surface adverse effects (including dry eye) in patients receiving PD‐L1 inhibitors, though this may be confounded by older age in that subgroup
Hamid et al. (2013)	Phase 1 expansion cohort (non‐randomized)	135 (melanoma)	Lambrolizumab (anti‐PD‐1)	Four cases of DED reported occurring in at least 1% of the treated population	Phase 1 clinical trial safety reporting; adverse events were collected systematically using CTCAE v4.0 criteria	Unspecified	Unspecified	Uveitis *n* = 2 (1.5%), Visual impairment *n* = 2 (1.5%)
Hodi et al. (2014)	Phase 1 dose‐escalation trial	46 (melanoma)	Ipilimumab + bevacizumab	Two cases of DED reported in the first 12 weeks after treatment	Phase 1 trial safety monitoring for the combination of ipilimumab and bevacizumab. Adverse events were graded using CTCAE v3.0	Unspecified	Unspecified	Uveitis (4.3%, 2/46)
Mazharuddin et al. (2022)	Retrospective cohort (single centre)	1280 patients (130 with ocular AEs)	Nivolumab, pembrolizumab, atezolizumab, ipilimumab, durvalumab, avelumab and ipilimumab/nivolumab	Corneal toxicity (31% of ocular AEs), with dry eye syndrome as the most common specific AE (15%, 28/180 events)	Retrospective review of medical records for patients on ICIs who underwent ocular examination by an optometrist or ophthalmologist	Clinical diagnosis made by eye care provider during examination	Based on standard clinical evaluation; no protocol‐defined objective testing. DED grading was based per CTCAE (most dry eye cases were Grade 1)	Neuro‐ophthalmic (14%), uveitis/scleritis (13%), retinopathy (13%) and periocular disorders (11%)
Noble et al. (2020)	Retrospective case series (3 centres)	11 patients	Pembrolizumab, ipilimumab, nivolumab, durvalumab and combination therapy	One patient (9%) developed dry eye syndrome and blepharitis	Retrospective chart review at three tertiary ophthalmology clinics; patients were identified via search for ICI terms in medical records (2000–2017)	Clinical diagnosis based on symptoms (foreign body sensation, redness and blurry vision) and examination findings (blepharitis, superficial punctate keratitis)	Standard comprehensive ophthalmic examination by ophthalmologist; no specific objective dry eye tests	Anterior uveitis, panuveitis and VKH‐like reactions. Most cases are managed without discontinuing ICIs
Ramos‐Casals and Fisher (2019)	Retrospective database analysis (ICIR)	26 (11 women and 15 men)	Nivolumab (*n* = 9), Pembrolizumab (*n* = 7), Durvalumab (*n* = 4) and Nivolumab/Ipilimumab (*n* = 6)	17/26 (65%) reported dry eye symptoms. 10/16 (62%) had abnormal ocular tests (e.g. Schirmer test ≤5 mm). Severe symptoms (e.g. photophobia) in some cases	Based on clinical suspicion of Sjögren's syndrome in registry patients	Ocular tests performed per European Community Study Group recommendations (specific tests not detailed, but results classified as abnormal/normal)	Patient‐reported dry eye symptoms	Conjunctivitis (not quantified)
Warner et al. (2019)	Prospective cohort study	20 (14 male, six female) with metastatic melanoma (*n* = 10), metastatic carcinoma (*n* = 6) and recurrent respiratory papillomatosis (*n* = 4)	Avelumab (*n* = 8), nivolumab (*n* = 5), pembrolizumab (*n* = 4), nivolumab/ipilimumab (*n* = 2) and M7824 (*n* = 1)	6/20 (30%) developed new dry eye symptoms. Schirmer's test ≤5 mm in 5/20 (25%) at evaluation. Symptoms ranged from mild to severe (e.g. photophobia). DED onset median: 70 days post‐ICI.	Prospective cohort evaluation of ICI‐treated patients with systematic ocular symptom assessment and objective testing.	Schirmer’s test (≤5 mm/5 minutes in ≥1 eye defined aqueous tear deficiency).	Unspecified. CTCAE v5 used for symptom severity used for grading	None reported beyond sicca syndrome
Weber et al. (2016)	Phase I/II trial	92 (ipilimumab‐refractory melanoma)	Nivolumab (±peptide vaccine)	3.3% (3/92) (grade 1–2)	Unspecified	Clinical trial AE reporting (CTCAE); ‘dry eye’ was captured as a treatment‐related adverse event among other listed toxicities in the safety analysis	No protocol‐driven ophthalmic‐specific testing	Blurred vision (1%)
Young et al. (2021)	Retrospective database analysis. The CTEP database	7727 (total exposed)	Nivolumab, Pembrolizumab, Atezolizumab and Durvalumab	0.6% prevalence of dry eye (likely underreported due to low‐grade events)	Retrospective database review of CTEP AE reports using CTCAE terms	In the serious AE subset, only 64% of patients with ocular AEs were evaluated by an ophthalmologist, indicating inconsistent specialist assessment	CTCAE grading applied by investigators (median grade 1 for routine AEs)	Uveitis, scleritis, keratitis and optic neuritis
Zimmer et al. (2016)	Retrospective cohort	496	Nivolumab and Pembrolizumab	1/496 patients (0.2%) reported ‘dry eyes’ as an ocular irAE	Retrospective multicentre review of anti‐PD‐1 therapy adverse events; ocular events were captured as part of a broader toxicity survey	Clinical diagnosis (‘dry eyes’) recorded by treating physicians (dermatologists/oncologists)	None: diagnosis likely based on patient symptoms and clinical impression. CTCAE grading applied (Grade 1 in the reported case) adopted as the grading criteria	Conjunctivitis, uveitis, blurred vision and iritis

Abbreviations: CTEP, Cancer Therapy Evaluation Program; FAERS, Food and Drug Administration Adverse Events Reporting System; ICIR, ImmunoCancer International Registry; RCT, randomized controlled trial.

### 3.3 Meta‐analysis

**FIGURE 2:**
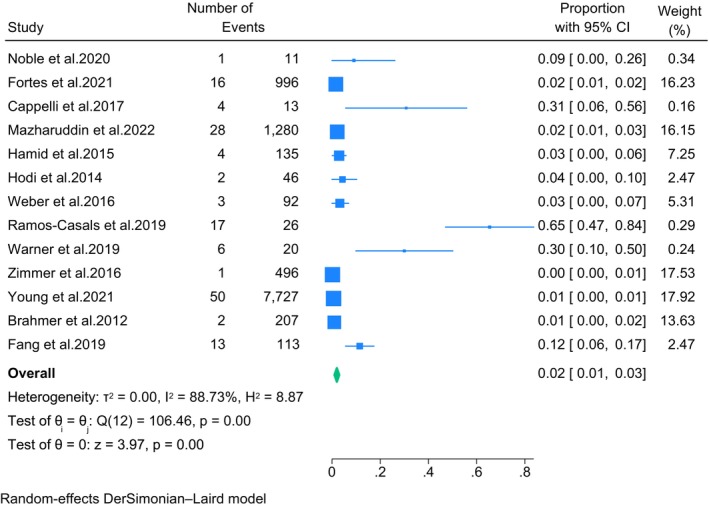
Incidence of dry eye disease (DED) in patients treated with immune checkpoint inhibitors (ICIs).

**FIGURE 3:**
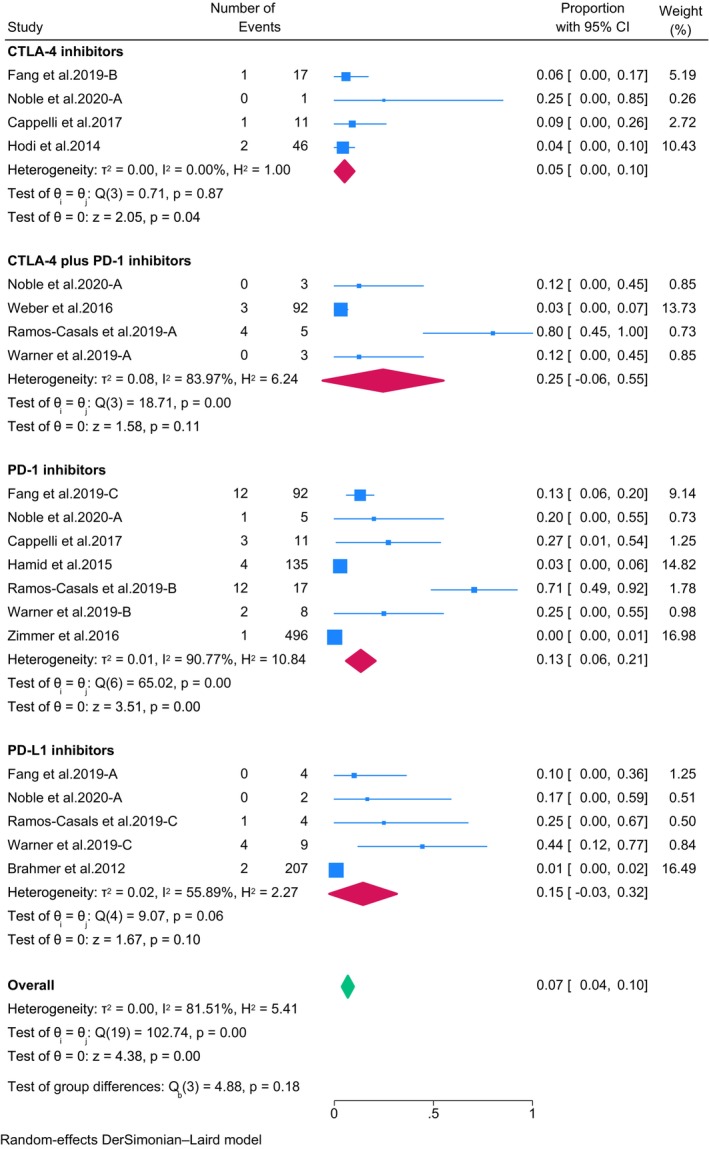
Incidence of dry eye disease (DED) stratified according to the type of immune checkpoint inhibitors (ICIs).

**FIGURE 4:**
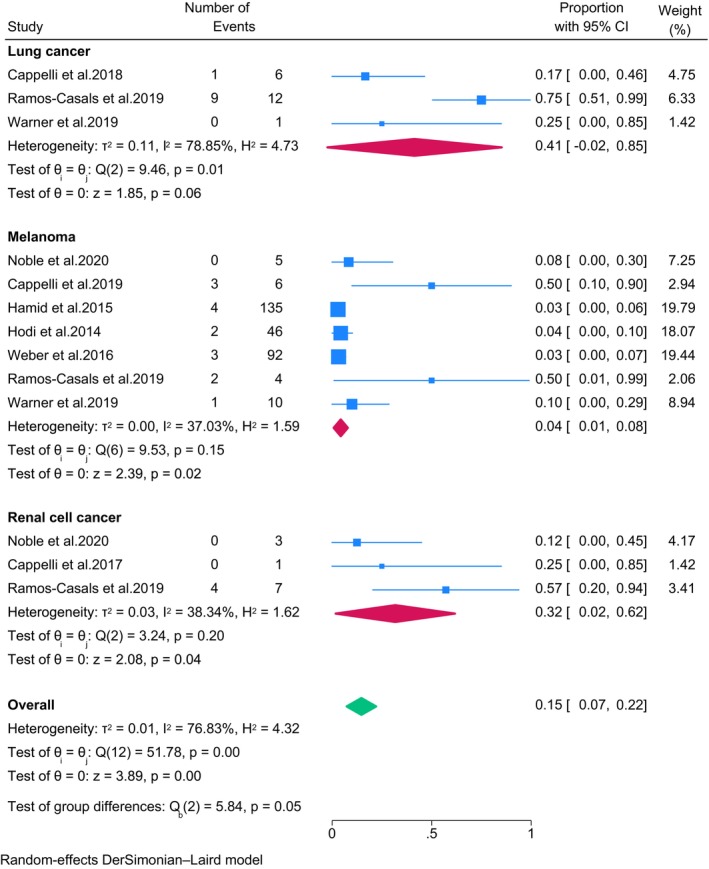
Incidence of dry eye disease (DED) stratified according to the type of tumors.

A one‐arm meta‐analysis assessed the incidence of DED in patients treated with ICIs. Data pooled from the included studies that had an extractable ICI‐exposed denominator revealed that the incidence of DED was 2% (95% CI: 1% to 3%), with the incidence across the pooled studies ranging from 0.2% to 65% (Figure 2). A subgroup analysis according to the type of ICI revealed that the incidence of DED was higher in patients treated with the combination of CTLA‐4 and PD‐1 inhibitors (25%) compared to those treated with CTLA‐4 inhibitors (5%), PD‐1 inhibitors (13%) or PD‐L1 inhibitors (15%) (Figure 3). However, the test for subgroup differences did not show a statistically significant difference (*p* = 0.18). This subgroup analysis was performed using the subset of studies with regimen‐specific extractable data. Similarly, a subgroup analysis according to the type of tumour showed that the incidence of DED was higher in lung cancer patients than in melanoma and renal cancer patients (41% vs 4% and 32%, respectively), with borderline evidence of subgroup differences (*p* = 0.05) (Figure 4); however, these subgroup estimates may be influenced by differences in study setting, referral pathways, and ascertainment intensity.

### 3.4 Publication bias

**FIGURE 5:**
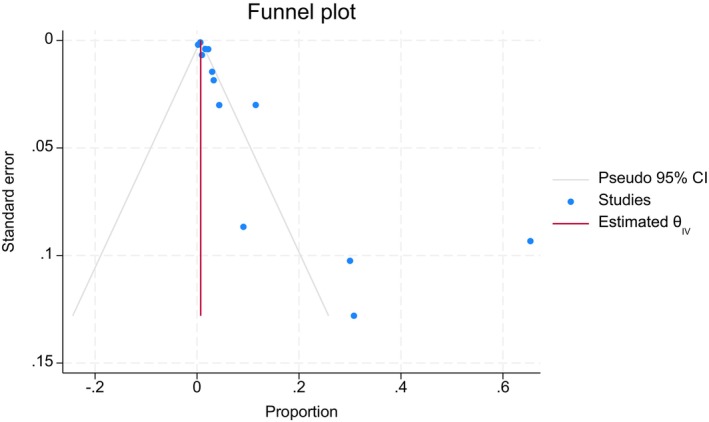
Funnel plot assessing publication bias for the 13 included studies.

Publication bias for the 13 included studies was assessed using a funnel plot as illustrated in Figure 5. The funnel plot depicts noticeable asymmetry, particularly with a clustering of smaller studies (those with larger standard errors) on the right‐hand side of the plot. This pattern is consistent with possible publication bias and/or small‐study effects, whereby smaller studies reporting higher proportions of dry eye may be more likely to appear in the published literature. However, given the substantial between‐study heterogeneity (I² = 89%) and the proportion‐based outcome, funnel plot asymmetry is not specific for publication bias and may also reflect clinical and methodological heterogeneity.

Conversely, the observed asymmetry may also be explained by the clinical and methodological heterogeneity across the included studies. Moreover, the divergent sample sizes range from as few as 11 patients to over 7000, and the ICIs used differ across studies, including nivolumab, pembrolizumab, atezolizumab, ipilimumab and various combinations. These differences likely influence the detection and reporting of DED, contributing to between‐study variation. Smaller studies may have focused on patients with higher risk or more severe symptoms, while larger trials or registry studies may underreport low‐grade or subclinical events. Additionally, different ICIs may have varying propensities to cause ocular side effects, further compounding the variability. The asymmetry in the funnel plot could reflect publication bias and underlying heterogeneity in study design, population and ICI regimen, all of which warrant consideration when interpreting the meta‐analytic findings. Therefore, the funnel plot findings should be interpreted cautiously and are considered hypothesis‐generating rather than definitive evidence of publication bias.

### 3.5 Sensitivity analysis

**FIGURE 6:**
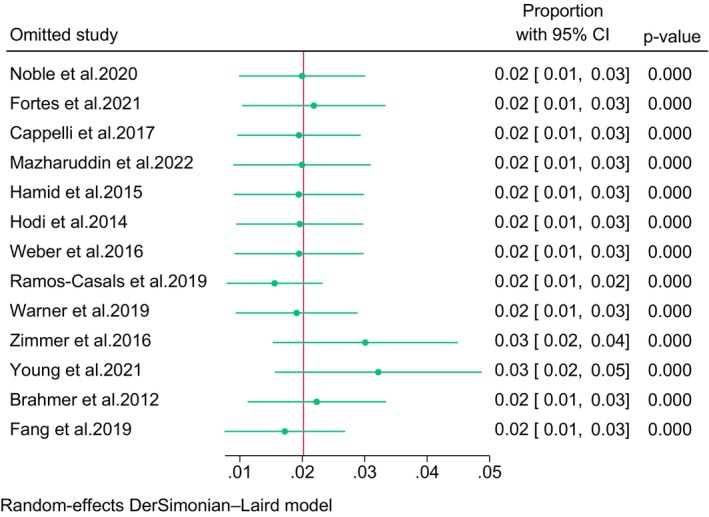
Leave‐one‐out sensitivity analysis assessing the impact of omitting individual studies on the pooled dry eye disease (DED) incidence estimate and its 95% confidence interval.

A leave‐one‐out sensitivity analysis was conducted to evaluate the effect of individual studies on the pooled incidence of DED among patients treated with ICIs. After sequentially omitting each study, the overall pooled incidence remained within approximately 2% to 3% with overlapping 95% CIs (Figure 6). None of the individual omissions materially altered the pooled estimate or its confidence interval, indicating that the overall result was not driven by any single study. The *p*‐values shown in the leave‐one‐out output reflect statistical testing against a null incidence of zero and are therefore not clinically informative; interpretation focuses on the stability of pooled estimates and confidence intervals. These findings support the robustness of the pooled incidence estimate despite substantial inter‐study heterogeneity.

### 3.6 Risk of bias assessment

All eight retrospective studies—ranging from single‐centre cohort analyses to large pharmacovigilance database evaluations—achieved satisfactory ratings across all three domains. Each study clearly defined the exposed cohort (patients receiving ICIs) and ensured appropriate case ascertainment of DED through clinical records or coded database outcomes in the selection domain. *Comparability* was maintained in most studies through adjustment or stratification for basic demographic and clinical variables, such as cancer type or ICI regimen. The *outcome* domain was consistently strong across studies, with appropriate outcome definitions and follow‐up durations, although standardized DED diagnostic criteria were not uniformly applied. Overall, the retrospective studies demonstrated a low risk of bias as illustrated in Table 2.

**TABLE 2:** Methodological quality assessment using Newcastle–Ottawa Scale (NOS) for retrospective studies.

Study	Selection	Comparability	Outcome	Overall quality
Fang et al. (2019)	****	**	**	Low risk
Noble et al. (2020)	****	**	**	Low risk
Fortes et al. (2021)	****	**	**	Low risk
Cappelli et al. (2017)	****	**	**	Low risk
Mazharuddin et al. (2022)	****	**	**	Low risk
Ramos‐Casals et al. (2019)	****	**	**	Low risk
Zimmer et al. (2016)	****	**	**	Low risk
Young et al. (2021)	****	**	**	Low risk

*Note*: Asterisks denote Newcastle Ottawa Scale points. Each asterisk represents one point. Maximum domain scores are Selection 4, Comparability 2, Outcome 3 (total 0 to 9).

On the other hand, three non‐randomized interventional trials—comprising early‐phase clinical studies and open‐label dose‐escalation trials—were assessed using the ROBINS‐I tool (Brahmer et al., 2012; Hodi et al., 2014). These studies were evaluated across seven bias domains: potential confounding, participant selection, intervention classification, bias due to deviations from intended interventions, bias due to missing data, bias in measurement of outcomes and bias in selection of the reported result. All three studies demonstrated a moderate overall risk of bias, primarily driven by concerns in two key areas: *bias due to confounding* and *bias in measuring outcomes*.

Bias due to confounding was rated as moderate in all three trials due to the absence of control groups or adjustments for potential confounders such as prior ocular history, systemic autoimmune disease or concurrent therapies. Although participant selection and bias in classification of interventions were reported and judged at low risk, limitations in outcome measurement—particularly reliance on subjective symptoms without standardized ophthalmologic assessment—were evident. Nevertheless, adherence to interventions was well maintained, and outcome reporting was complete and transparent across studies. Table 3 shows that while the non‐randomized trials contributed valuable safety and tolerability data, their findings should be interpreted cautiously regarding causality. Additional prospective observational studies or controlled trials are warranted to better characterize the risk of DED associated with specific ICI regimens.

**TABLE 3:** Risk of bias assessment using Risk Of Bias In Non‐randomized Studies of Interventions (ROBINS‐I) for non‐randomized studies of interventions.

Study	Confounding	Selection of participants	Classification of interventions	Deviations from intended interventions	Missing data	Measurement of outcomes	Reporting bias	Overall risk
Hamid et al. (2013)	Moderate	Low	Low	Low	Low	Moderate	Low	Moderate
Hodi et al. (2014)	Moderate	Low	Low	Low	Low	Moderate	Low	Moderate
Brahmer et al. (2012)	Moderate	Low	Low	Low	Low	Moderate	Low	Moderate

Further, the single randomized controlled trial included in this review was assessed using the Cochrane Risk of Bias 2 (RoB 2) tool and was judged to have a low risk of bias across all assessed domains (Table 4) (Weber et al., 2016). The study employed a robust randomization process, clearly reported allocation concealment, and ensured minimal deviations from the intended intervention. The *outcome data* were complete, and the methods for measuring DED, although not detailed with ophthalmologic specificity, were consistently applied across study arms.

**TABLE 4:** Risk of bias assessment using Risk of Bias 2 (RoB 2) for randomized controlled trial.

Study	Randomization	Deviations from intervention	Missing data	Measurement of outcome	Reporting bias	Overall risk
Weber et al. (2016)	Low	Low	Low	Low	Low	Low

## 4 DISCUSSION

This systematic review and meta‐analysis provides the first comprehensive synthesis of evidence on DED incidence in patients treated with ICIs. Pooled data from the 13 included studies, encompassing 11 184 patients, showed a pooled DED estimate of 2% (95% CI: 1%–3%). Although the pooled incidence of DED was modest, the range across individual studies was wide (0.2% to 65%), highlighting substantial variability across the 13 included studies. This variability is attributed to the heterogeneity observed in the included studies' methodologies.

The wide variability in reported DED incidence (0.2%–65%) likely reflects both the multifactorial nature of the condition and significant methodological heterogeneity across the included studies. Notably, a higher incidence of DED was observed in studies detailing combination therapies, such as CTLA‐4 and PD‐1 inhibitors (Noble et al., 2020; Ramos‐Casals et al., 2019; Weber et al., 2016), compared to monotherapies; however, the test for subgroup differences was not statistically significant (*p* = 0.18).

Several key factors may explain the observed variability. First, differences in patient populations across studies—including cancer type (e.g. lung vs melanoma), baseline risk of dry eye, pre‐existing autoimmune conditions, and demographic characteristics—could influence susceptibility to ICI‐induced DED. Second, heterogeneity in treatment regimens, such as variations in ICI drug class (anti‐PD‐1, anti‐PD‐L1, and anti‐CTLA‐4), use of monotherapy versus combination therapy and duration of treatment may contribute to differences in incidence.

Third, and perhaps most substantially, methodological inconsistency in defining and detecting DED likely played a central role. Studies employing protocol‐driven ophthalmologic assessments, such as Schirmer’s testing, tended to identify higher DED rates (e.g. Ramos‐Casals et al., 2019; Warner et al., 2019), whereas large registry/database studies relying on passive reporting likely undercaptured low‐grade cases. In contrast, large‐scale database analyses relying on passive or nonspecific adverse event reporting (e.g. Young et al., 2021) likely captured only more severe or clinically apparent cases, thereby underestimating the true prevalence. Finally, variability in follow‐up duration across studies may have influenced detection, particularly for late‐onset DED.

Secondly, we posit that the mechanism of ICI‐induced DED may involve immune‐mediated lacrimal gland dysfunction or ocular surface inflammation, as suggested in cases of concurrent sicca syndrome by Cappelli et al. (2017) and abnormal Schirmer's tests (Ramos‐Casals et al., 2019). In this light, this review postulates that ICIs may trigger a de novo autoimmune exocrinopathy, wherein T‐cell activation, which goes unchecked due to PD‐1 or CTLA‐4 inhibition, targets lacrimal and salivary tissues (Fenech et al., 2025; Ileana Dumbrava et al., 2018). Similarly, Ramos‐Casals et al. documented abnormal Schirmer's test results (≤5 mm) in 62% of ICI‐treated patients with DED symptoms, indicating objective tear deficiency (Ramos‐Casals et al., 2019). Collectively, these findings point to lacrimal gland hypofunction as a key driver of ICI‐induced DED, distinct from evaporative dry eye caused by meibomian gland dysfunction (Sheppard & Nichols, 2023).

From a molecular standpoint, the incidence of DED in patients treated with ICIs can be explained through the breakdown of immune tolerance. Conventionally, PD‐1 and CTLA‐4 receptors on T‐cells act as ‘brakes’ to prevent autoreactivity, particularly in tissues with high immune privilege (e.g. the eye) (Rajagopal et al., 2024). By blocking these checkpoints, ICIs unleash cytotoxic T‐cells and Th17‐mediated responses, which may infiltrate the lacrimal gland or ocular surface, disrupting tear production and stability. Animal models of PD‐1 deficiency develop lymphocytic infiltration of lacrimal glands and reduced tear secretion, paralleling human observations (Zhu et al., 2022).

Additionally, elevated pro‐inflammatory cytokines (e.g. IL‐17 and IFN‐γ) in the tear film of ICI‐treated patients, akin to those seen in Sjögren's syndrome as postulated by Li et al. (2024) and Kang et al. (2011), could perpetuate ocular surface damage through goblet cell loss and corneal epithelial barrier dysfunction (Brown et al., 2024). Notably, the incidence of DED with combination ICIs (25% two‐sided estimate in this analysis) may reflect amplified immune dysregulation, as CTLA‐4 inhibition promotes T‐cell priming in lymphoid organs, while PD‐1 blockade exacerbates tissue‐specific effector responses.

However, critical knowledge gaps remain. First, it is unclear why only a subset of patients develops DED despite uniform ICI exposure, suggesting contributions from genetic predisposition (e.g. HLA subtypes) or pre‐existing subclinical autoimmunity (Higuchi et al., 2021; Muñiz‐Castrillo et al., 2020). Second, the role of B‐cells and autoantibodies (e.g. anti‐muscarinic receptor antibodies) in ICI‐induced DED warrants investigation (Ghosh et al., 2022), as these are implicated in traditional autoimmune dry eye but were absent in Cappelli et al.'s cohort (2017). Third, the reversibility of lacrimal gland damage post‐ICI cessation is poorly characterized; some studies report symptom resolution with topical therapy, while others describe chronicity. Prospective studies incorporating histopathological correlates (e.g. lacrimal gland biopsies) and tear cytokine profiling could clarify these mechanisms and identify biomarkers for early intervention.

Lung cancer patients exhibited a numerically higher incidence (41%) than melanoma (4%) or renal cancer (32%) cohorts, but this trend did not reach significance (*p* = 0.05). This observation raises important questions about the tumour‐intrinsic and treatment‐related factors that may predispose certain patients to ocular toxicity. Importantly, these apparent subgroup differences may reflect not only tumour‐ or regimen‐specific biology, but also systematic differences in surveillance intensity, access to ophthalmic assessment, and referral enrichment across cohorts, which may materially influence observed event rates. Nonetheless, several hypotheses may explain this variability. Firstly, lung cancer is associated with a more inflamed tumour microenvironment (TME) characterized by higher baseline immune infiltration, PD‐L1 expression and pre‐existing autoantibodies, as illustrated by Wu, Wang, et al. (2024), compared to melanoma or renal cell carcinoma. This observation is hypothesis‐generating; lung cancer cohorts may show higher observed rates due to tumour‐ or regimen‐related factors and systematic differences in surveillance intensity and referral enrichment. In contrast, while highly responsive to ICIs, melanoma may involve different T‐cell activation patterns (e.g. more systemic rather than mucosal tissue targeting), potentially explaining the lower reported DED incidence.

Moreover, the low reported DED incidence in melanoma (4%) may reflect underdetection due to prioritization of life‐threatening irAEs (e.g. colitis and pneumonitis) in clinical trials, with ocular symptoms overlooked or attributed to non‐immune causes. Collectively, these findings align with pre‐existing reports on the incidence of the ICI‐related spectrum of immune‐related adverse events (irAEs), including ophthalmic manifestations such as dry eye, uveitis, and scleritis (Ameri et al., 2024; Chang et al., 2025). While findings from this analysis warrant cautious interpretation based on the wide confidence intervals and limited sample sizes in some subgroups, overall, they highlight the need for standardized diagnostic protocols and proactive monitoring of ocular surface toxicity in ICI‐treated patients, particularly those receiving a combination regimen or with pre‐existing risk factors.

### 4.1 Strengths and limitations

The strengths of this meta‐analysis include adherence to PRISMA guidelines, rigorous risk‐of‐bias assessment and comprehensive subgroup analyses. However, this meta‐analysis has several important limitations when interpreting the results. First, the predominance of retrospective studies (8 of 13 included studies) introduces potential selection and reporting biases, as these designs often rely on incomplete medical records or passive surveillance systems. For example, extensive database analyses, such as Young et al. [*n* = 7727] (2021) and Mazharuddin et al. [*n* = 1280] (2022), likely underestimated mild DED cases, as such real‐world datasets prioritize severe adverse events (AEs). Conversely, smaller studies (e.g. Noble et al. [*n* = 11] (2020) and Cappelli et al. [*n* = 13] (2017)) may overrepresent symptomatic or severe DED due to their limited sample sizes and focus on specialized populations.

Second, significant heterogeneity in how DED was defined and reported across studies complicates the interpretation of pooled incidence estimates. While some trials (e.g. Weber et al. (2016)) explicitly listed DED as an AE, others (e.g. Brahmer et al., 2012; Zimmer et al., 2016) grouped it within broader ‘ocular AEs’ without specific quantification. This inconsistency suggests our meta‐analysis may not fully capture the actual burden of ICI‐induced DED. Additionally, several studies (e.g. Cappelli et al., 2017; Hodi et al., 2014; Warner et al., 2019) focused primarily on systemic or rheumatic immune‐related AEs, with ocular toxicity reported only incidentally, further contributing to variability in detection and reporting. Additionally, the variations in diagnostic methods might be another key source of heterogeneity.

Third, the timing and duration of follow‐up varied widely across studies. For instance, Ramos‐Casals & Fisher (Ramos‐Casals et al., 2019) reported a median DED onset of 6.5 months post‐ICI initiation, underscoring the need for more extended follow‐up periods in prospective studies, as many clinical trials prioritize early‐onset toxicities. Moreover, the underrepresentation of Asian and paediatric populations in the included studies limits the generalizability of our findings, as these groups may have distinct risk profiles for ocular surface disease.

Finally, while publication bias was evident in our funnel plot analysis, the clinical implications of this bias are nuanced. Smaller studies with higher DED incidence estimates (e.g. Ramos‐Casals & Fisher [65%]) were overrepresented compared to larger, more conservative analyses. This skew may reflect true clinical heterogeneity (e.g. differences in ICI regimens or patient populations) and methodological gaps (e.g. lack of standardized ocular assessments). Prospective studies with protocolized ophthalmic evaluations and diverse population inclusion are needed to refine incidence estimates and optimize management strategies.

## 5 CONCLUSIONS

This systematic review and meta‐analysis provides the first pooled estimate of DED incidence in patients treated with ICIs. Clinically, the available evidence indicates that ICI‐associated DED is frequently mild and can often be managed with topical therapy without interruption of anticancer treatment. Nevertheless, a minority of patients may develop clinically significant symptoms necessitating escalation of therapy, including systemic immunomodulatory treatment, particularly in referral‐enriched cohorts or in the context of concurrent sicca features. These observations support the clinical relevance of proactive ocular symptom surveillance, although the frequency of severe DED in routine oncology practice cannot be precisely inferred from the present pooled estimates. This parallels management strategies for other irAEs, where early recognition and local treatment preserve cancer therapy continuity. However, the lack of standardized grading (e.g. CTCAE vs clinician‐reported scales) and long‐term follow‐up data in included studies limit conclusions about chronicity or recurrence. Future studies should incorporate prospective ocular surface evaluations (e.g. tear film osmolarity, corneal staining) to stratify risk and optimize interventions.

As ICIs transform the oncology landscape, recognizing and proactively managing immune‐related ocular toxicities, including DED, should be integrated into routine oncologic care. Ophthalmologic surveillance—particularly in high‐risk patients or those receiving combination regimens—may improve early detection, prevent progression, and enhance treatment adherence. Future studies should incorporate uniform diagnostic criteria and patient‐reported outcomes to ensure a more accurate understanding of the burden and impact of DED in this expanding patient population.

## AUTHOR CONTRIBUTIONS

K.‐Y. C. contributed to conceptualization, methodology, software, investigation, validation, writing the original draft, visualization and formal analysis. H.‐C. C. was responsible for conceptualization, methodology and software. C.‐M. C. handled methodology, investigation, validation, supervision and project administration. All authors reviewed the manuscript.

## FUNDING INFORMATION

No specific funding was received from any funding bodies in the public, commercial or not‐for‐profit sectors to conduct the work described in this manuscript.

## ETHICS APPROVAL AND CONSENT TO PARTICIPATE

Not applicable. This study does not involve human participants, human data or human tissue.

## CONSENT FOR PUBLICATION

Not applicable. This study does not involve individual data.

## Data Availability

The datasets used and analysed during the current study are available from the corresponding author on reasonable request.

## APPENDIX A Full search strategy

**Table:**

Database	Search strategy
PubMed	(‘epidemiology’[MeSH Subheading] OR ‘epidemiology’[All Fields] OR ‘incidence’[All Fields] OR ‘incidence’[MeSH Terms] OR ‘incidences’[All Fields] OR ‘incident’[All Fields] OR ‘incidents’[All Fields]) AND (‘dry eye syndromes’[MeSH Terms] OR (‘dry’[All Fields] AND ‘eye’[All Fields] AND ‘syndromes’[All Fields]) OR ‘dry eye syndromes’[All Fields] OR (‘dry’[All Fields] AND ‘eye’[All Fields] AND ‘disease’[All Fields]) OR ‘dry eye disease’[All Fields]) AND (‘patient s’[All Fields] OR ‘patients’[MeSH Terms] OR ‘patients’[All Fields] OR ‘patient’[All Fields] OR ‘patients s’[All Fields]) AND (‘therapy’[MeSH Subheading] OR ‘therapy’[All Fields] OR ‘treat’[All Fields] OR ‘treating’[All Fields] OR ‘treated’[All Fields] OR ‘treats’[All Fields]) AND (‘immune checkpoint inhibitors’[Pharmacological Action] OR ‘immune checkpoint inhibitors’[Supplementary Concept] OR ‘immune checkpoint inhibitors’[All Fields] OR ‘immune checkpoint inhibitors’[MeSH Terms] OR (‘immune’[All Fields] AND ‘checkpoint’[All Fields] AND “inhibitors’[All Fields] OR ‘PD‐1’[All Fields] OR ‘pd l1’[All Fields])
Cochrane Library	(MeSH descriptor: [Immune Checkpoint Inhibitors] OR ‘immune checkpoint inhibitor*’:ti,ab,kw OR ‘checkpoint inhibitor*’:ti,ab,kw OR ‘programmed cell death protein 1’:ti,ab,kw) AND (MeSH: [Dry Eye Syndromes] OR ‘dry eye disease’:ti,ab,kw OR ‘dry eye’:ti,ab,kw OR ‘keratoconjunctivitis sicca’:ti,ab,kw) AND (‘immune‐related adverse event*’:ti,ab,kw OR irAE*:ti,ab,kw OR ‘ocular adverse event*’:ti,ab,kw )
Web of Science	TS=( (‘immune checkpoint inhibitor*’ OR ‘programmed cell death protein 1’ OR PD‐1 OR PD1 OR ‘programmed death‐ligand 1’ OR PD‐L1 OR PDL1 OR ‘cytotoxic T‐lymphocyte associated protein 4’ OR CTLA‐4 OR CTLA4) ) AND TS=( (‘dry eye disease’ OR ‘dry eye’ OR ‘keratoconjunctivitis sicca’) ) AND TS = ( (‘immune‐related adverse event*’ OR irAE* OR ‘ocular adverse event*’ OR ‘ocular toxicit*’) )

Abbreviations: ab, abstract; kw, keywords; TS, topic search; ti, title; *, singular or plural capture.
